# Reply to the ‘Comment on “Improving the efficiency of a CIGS solar cell to above 31% with Sb_2_S_3_ as a new BSF: a numerical simulation approach by SCAPS-1D”’ by A. Kirk, *RSC Adv.*, 2024, https://doi.org/10.1039/D4RA03002H

**DOI:** 10.1039/d4ra05885b

**Published:** 2024-10-24

**Authors:** Md. Ferdous Rahman, Mithun Chowdhury, Latha Marasamy, Mustafa K. A. Mohammed, Md. Dulal Haque, Sheikh Rashel Al Ahmed, Ahmad Irfan, Aijaz Rasool Chaudhry, Souraya Goumri-Said

**Affiliations:** a Advanced Energy Materials and Solar Cell Research Laboratory, Department of Electrical and Electronic Engineering, Begum Rokeya University Rangpur 5400 Bangladesh ferdousapee@gmail.com; b Facultad de Química, Materiales-Energía, Universidad Autónoma de Querétaro (UAQ), Santiago de Querétaro Querétaro C.P. 76010 Mexico; c College of Remote Sensing and Geophysics, Al-Karkh University of Science Al-Karkh Side, Haifa St. Hamada Palace Baghdad 10011 Iraq; d Department of Electronics and Communication Engineering, Hajee Mohammad Danesh Science and Technology University Dinajpur 5200 Bangladesh; e Department of Electrical, Electronic and Communication Engineering, Pabna University of Science and Technology Pabna 6600 Bangladesh; f Department of Chemistry, College of Science, King Khalid University P.O. Box 9004 Abha 61413 Saudi Arabia; g Department of Physics, College of Science, University of Bisha P.O. Box 551 Bisha 61922 Saudi Arabia; h Physics Department, Colleges of Science and General Studies, Alfaisal University P.O. Box 50927 Riyadh 11533 Saudi Arabia sosaid@alfaisal.edu

## Abstract

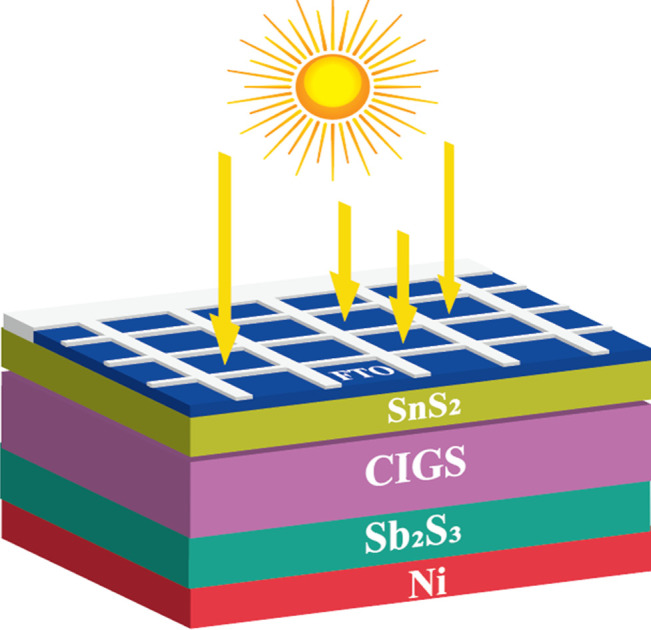

## Our Reply for Alexander P. Kirk comment

**Fig. 1:**
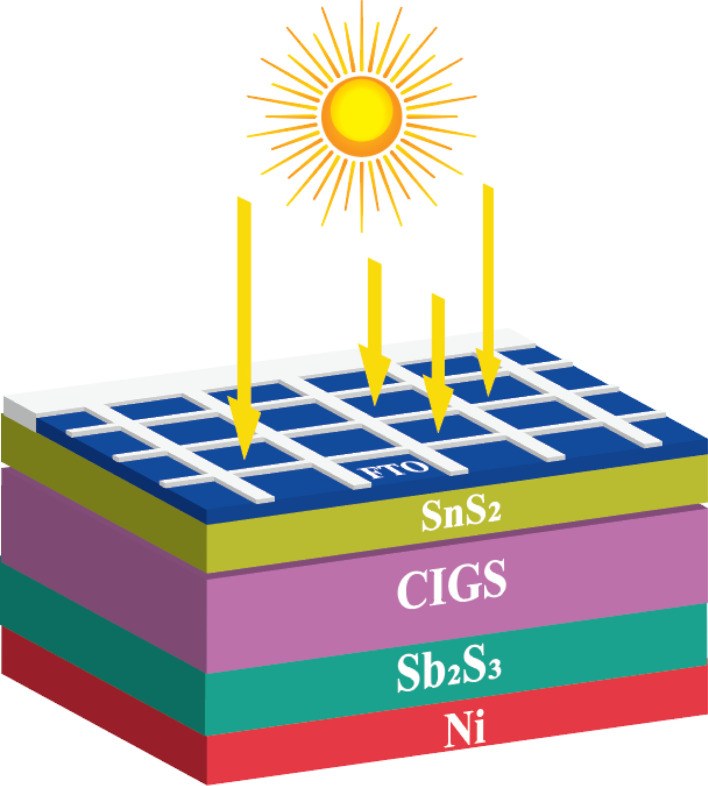
Proposed CIGS solar cell with Sb_2_S_3_ BSF layer.

We sincerely appreciate the thoughtful feedback on our manuscript (https://doi.org/10.1039/D3RA07893K). In the comment, Alexander P. Kirk has referenced a reported efficiency of 40.70% for our solar cell design. However, we would like to clarify that the actual efficiency of our CIGS solar cell (Copper Indium Gallium Selenide) with the addition of a new BSF (back surface field) layer made from Sb_2_S_3_ (Antimony Sulfide) is 31.15%. When the BSF layer is not used, the efficiency is 22.14%.^1^ To ensure transparency and accuracy, these efficiency values have been clearly stated at multiple points throughout our manuscript. Specifically, the efficiency data is provided in the following sections: (i) Title, (ii) Abstract, (iii) Introduction, (iv) Results and discussion, (v) *J*–*V* parts, Table 1, and Table 2, and (vi) Conclusions in the reputed manuscript.^1^ By mentioning the efficiency values in multiple sections, we have taken steps to avoid any confusion and ensure clarity regarding the performance of our solar cell both with and without the BSF layer. In Fig. 1, we have shown the proposed CIGS solar cell with Sb_2_S_3_ BSF layer.

**Table 1:** PV performance of suggested cell compared to other reported CIGS solar cell without BSF

Types of research	CIGS layer thickness (μm)	*V*_OC_ (V)	*J*_SC_ (mA cm^−2^)	FF (%)	*η* (%)	Ref.
Experimental	2.0	0.671	34.90	77.60	18.10	2
Experimental	1.0	0.689	35.71	78.12	19.20	3
Experimental	2.2	0.690	35.50	81.20	19.90	4
Experimental	—	0.741	37.80	80.60	22.60	5
Theoretical	1.0	0.743	34.47	83.09	21.30	6
**Theoretical**	**1.0**	**0.91**	**28.21**	**86.31**	22.14* (without BSF)	*This work

**Table 2:** Impact of BSF layer in comparison with related research

Types of research	Absorber	BSF	*η* without BSF (%)	*η* with BSF (%)	Ref.
Experimental	Si	ZnS	6.40	11.02	7
Experimental	Si	Al	12.96	13.75	8
Experimental	CIGS	MoSe_2_	9	14	9
Theoretical	CdTe	V_2_O_5_	19.58	23.50	10
Theoretical	CZTS	CZTS	12.05	14.11	11
Theoretical	ZnTe	Sb_2_Te_3_	7.14	18.33	12
Theoretical	CZTSSe	SnS	12.30	17.25	13
Theoretical	CIGS	Si	16.39	21.30	6
Theoretical	CIGS	μc-Si : H	19.80	23.42	14
Theoretical	CIGS	SnS	17.99	25.29	15
Theoretical	CIGS	PbS	22.67	24.22	16
**Theoretical**	**CIGS**	**Sb**_**2**_ **S**_**3**_	22.14*	31.15*	*This work

In contrast to the comment, I have utilized all the optimized parameters listed in Tables 3 and 4 for our proposed solar cell structure (FTO/SnS_2_/CIGS/Sb_2_S_3_/Ni) in the SCAPS-1D simulation. To determine the optimal absorber thickness, we conducted an extensive analysis, varying the thickness from 250 nm to 3000 nm. Across this range, the power conversion efficiency of our proposed structure varied from 19.80% to a maximum of 40.70%. It is important to note that the 40.70% efficiency does not represent the optimized efficiency for the solar cell. After a thorough investigation, we identified that an absorber thickness of 1 μm (1000 nm) is optimal. This specific thickness, as shown in Tables 3 and 4,^1^ provided efficiencies of 31.15% when using the Sb_2_S_3_ BSF layer and 22.14% without it. Therefore, the optimized efficiency with the 1 μm absorber is significantly lower than the 40.70% figure mentioned, which is the highest efficiency obtained during the range of testing but not the optimal one.

**Table 3:** Layer properties used in Al/FTO/SnS_2_/CIGS/Sb_2_S_3_/Ni solar cell^a^^17–20^

Parameters (unit)	FTO	SnS_2_	CIGS	Sb_2_S_3_
Layer type	Window	ETL	Absorber	BSF
Conductivity type	n^+^	n	p	P^+^
Thickness (μm)	0.05	0.05	1.0*	0.2
Bandgap (eV)	3.6	2.24	1.1	1.62
Electron affinity (eV)	4	4.24	4.2	3.70
Dielectric permittivity (relative)	9	10	13.6	7.08
CB effective DOS (cm^−3^)	2.2 × 10^18^	2.2 × 10^18^	2.2 × 10^18^	2.0 × 10^19^
VB effective DOS (cm^−3^)	1.8 × 10^19^	1.8 × 10^19^	1.8 × 10^19^	1.0 × 10^19^
Electron thermal velocity (cm s^−1^)	1 × 10^7^	1 × 10^7^	1 × 10^7^	1 × 10^7^
Hole thermal velocity (cm s^−1^)	1 × 10^7^	1 × 10^7^	1 × 10^7^	1 × 10^7^
Electron mobility (cm^2^ V^−1^ s^−1^)	100	50	100	9.8
Hole mobility (cm^2^ V^−1^ s^−1^)	25	50	25	10
Donor density, *N*_D_ (cm^−3^)	1 × 10^18^	1 × 10^15^	0	0
Acceptor density, *N*_A_ (cm^−3^)	0	0	1 × 10^16^*	1 × 10^15^
Defect type	SA	SA	SD	SD
Defect density (cm^−3^)	1 × 10^12^	1 × 10^12^	1 × 10^12^	1 × 10^12^

a SA single acceptor, SD single donor, (*) variable field.

**Table 4:** Interface factors used in Al/FTO/SnS_2_/CIGS/Sb_2_S_3_/Ni solar cell

Parameters (unit)	Sb_2_S_3_/CIGS interface	CIGS/SnS_2_ interface
Defect type	Neutral	Neutral
Electron capture cross-section, *σ*_e_ (cm^2^)	1 × 10^19^	1 × 10^19^
Hole capture cross-section, *σ*_p_ (cm^2^)	1 × 10^19^	1 × 10^19^
Defect position above the highest *E*_V_ (eV)	0.06	0.06
Interface defect density (cm^−2^)	1 × 10^12^	1 × 10^12^

Additionally, Alexander P. Kirk raised concerns regarding our consideration of hot carrier collection in the manuscript. However, it is crucial to highlight that in Tables 3 and 4, we have presented all the optimized parameters used in our SCAPS-1D simulation, which includes all relevant factors for accurately simulating the performance of our solar cell structure. The results are reflective of the carefully optimized conditions, and hot carrier collection was not an assumed factor in our analysis. By clarifying the distinction between the highest and optimized efficiencies and addressing the concerns about parameter usage, we ensure that the results and methods presented are accurate and consistent with the scope of the study.

## Ethical approval

The all authors declare that the manuscript does not have studies on human subjects, human data or tissue, or animals.

## Data availability

Data will be available on request.

## Conflicts of interest

The authors have no conflicts of interest.
